# Establishment and validation of the prediction model for postoperative delirium risk factors in older patients after total knee arthroplasty: A retrospective study

**DOI:** 10.1097/MD.0000000000038745

**Published:** 2024-06-28

**Authors:** Ling-Xia Song, Yi Qin, Li Yang, Ding-Bi Xing, Ying Li, Fu-Qi Lei, Lian-Hong Wang

**Affiliations:** a Department of Orthopedics, Affiliated Hospital of Zunyi Medical University, Zunyi, China; b Department of Nursing, Affiliated Hospital of Zunyi Medical University, Zunyi, China; c Department of Nursing, Zunyi Medical University, Zunyi, China.

**Keywords:** nomogram, postoperative delirium, prediction model, risk factors, total knee arthroplasty

## Abstract

This study aimed to establish an effective predictive model for postoperative delirium (POD) risk assessment after total knee arthroplasty (TKA) in older patients. The clinical data of 446 older patients undergoing TKA in the Orthopedics Department of our University from January to December 2022 were retrospectively analyzed, and the POD risk prediction model of older patients after TKA was established. Finally, 446 patients were included, which were divided into training group (n = 313) and verification group (n = 133). Logistic regression method was used to select meaningful predictors. The prediction model was constructed with nomographs, and the model was evaluated with correction curve and receiver operating characteristic curve. The logistic regression analysis showed that age, educational level, American Society of Anesthesiologists grade, accompaniment of chronic obstructive pulmonary disease, accompaniment of cerebral stroke, postoperative hypoxemia, long operation time, and postoperative pain were independent risk factors for POD after TKA (*P* < .05). The nomogram prediction model established. The area under receiver operating characteristic curve of the model group and the validation group were 0.954 and 0.931, respectively. The calibration curve of the prediction model has a high consistency between the 2 groups. The occurrence of POD was associated with age, educational level, American Society of Anesthesiologists grade, accompaniment of chronic obstructive pulmonary disease, accompaniment of cerebral stroke, postoperative hypoxemia, long operation time, and postoperative pain in TKA patients.

## 1. Introduction

Total knee arthroplasty (TKA) is the most commonly used method for the treatment of end-stage knee osteoarthritis, rheumatoid arthritis, and other diseases in older patients, which can effectively improve the joint function, relieve joint pain, and improve the quality of life of patients.^[1]^ Due to the reduced brain function and surgical trauma, postoperative delirium (POD) has become a common complication in older patients.^[2,3]^ POD is an acute brain syndrome characterized by attention, orientation, and cognitive impairment, mainly occurring within 1 to 3 days after surgery, and its incidence is 14.8% to 62%.^[4,5]^ In addition, POD can increase the time of hospital stay, cost of hospitalization, and the physical, psychological, and economic burdens on patients, and it may even increase the risk of death, thereby imposing substantial agonies and financial burdens to patients and their families.^[6,7]^ Previous studies have shown that 30% to 40% of POD is preventable, while the prerequisite for effective prevention requires the identification of high-risk POD patients and the subsequent implementation of targeted interventions.^[8]^ At present, the risk prediction model is widely used to identify high-risk groups, which can rapidly and effectively predict and identify the cases by clarifying the risk factors of related diseases, and it has high clinical applicability. Therefore, the present study aimed to develop a risk prediction model for POD in older patients after TKA, and further validate the effectiveness of this model, thereby assisting clinicians to evaluate the risk of delirium in such patients, and the early use of preventive measurements to improve the rehabilitation outcomes and the study population’s quality of life.

## 2. Information and methods

### 2.1. Data sources and data collection

POD was diagnosed according to the Diagnostic and Statistical Manual of Mental Disorders (5th edition) that was issued by the American Psychiatric Association (APA),^[9]^ based on the confusion assessment method (CAM) as follows: acute onset and fluctuating course; inattention; disorganized thinking; and altered level of consciousness. If patients met both the acute onset and fluctuating course and inattention, as well as disorganized thinking or altered level of consciousness simultaneously, the diagnosis of POD could be confirmed.^[4]^ The keywords in medical records were extracted, including “delirium,” “mental status change,” “agitation,” “restlessness,” “gibberish,” “inattention,” “disorientation,” and “hallucination.” Patients who were clearly diagnosed with POD in the medical records were categorized into the delirium group; patients who were not clearly diagnosed with POD, while delirium-related keywords were documented in the medical records, the medical records were documented and discussed by consultation with psychiatrists. Finally, patients confirmed with POD were assigned into the delirium group, and patients without POD were assigned into the non-delirium group. The study is approved by the Ethics Committee of Affiliated Hospital of Zunyi Medical University (KLL-2022-277). Results will be disseminated via peer-reviewed journal regardless of outcome.

### 2.2. Inclusion and exclusion criteria

Clinical data of patients who underwent TKA in the Department of Orthopedics of the Affiliated Hospital of Zunyi Medical University (Zunyi, China) from January to December 2022 were retrospectively analyzed. The inclusion criteria were as follows: age ≥ 60 years old; and undergoing TKA surgery for the first time. The exclusion criteria were as follows: involvement of malignant tumors, such as liver cancer and lung cancer; patients with severe liver or kidney diseases; utilizing antidepressant drugs or sedatives; patients with dementia or with the history of schizophrenia; incomplete data affecting the evaluation or ≥10% of data were missed.

### 2.3. Statistical analysis

SPSS 29.0 software (IBM, Armonk, NY) was used to perform the univariate and multivariate logistic regression analyses to screen the risk factors, and to compare statistical differences between the model group and validation group. Normally distributed quantitative data were described as *x**** ± ****s*, and were compared by the independent *t* test. Qualitative data were expressed as frequency and percentage (%), and compared by Kruskal–Wallis H test (χ^2^) test. The R 4.2.0 software was utilized for the development of the nomogram prediction model; the Bootstrap method was used for resampling for 1000 times for internal validation of the model, and the data of the verification group were used for the external validation. The receiver operating characteristic curve (ROC) and calibration curve were utilized to assess the discrimination capability and consistency of the model. *P* < .05 was considered statistically significant.

## 3. Results

### 3.1. General information

From January to December 2022, totally 479 patients who aged ≥ 60 years old underwent TKA in our hospital. Notably, 4 patients with malignant tumors, 2 patients who used antidepressants or sedatives, 1 patient with the history of schizophrenia, and 26 patients with incomplete medical records were excluded, in which data of 446 patients were finally included in the analysis. Patients were 7:3 randomized into the model group and verification group. Finally, data of 313 patients were used for the model establishment (POD occurred in 54 patients), and data of 133 patients were used for verification (POD occurred in 25 patients).

### 3.2. Risk factor analysis

#### 3.2.1. Univariate analysis

The occurrence of POD in older patients after TKA was closely associated with age, educational level, history of sleep disturbance, living style, preoperative hospital stay, hospital stay, American Society of Anesthesiologists (ASA) grade, accompaniment of diabetes, coronary heart disease, cerebral stroke, and chronic obstructive pulmonary disease (COPD), postoperative hypoxemia, preoperative albumin level, blood transfusion, operation time, and postoperative pain (*P* < .05) (Table 1).

**Table 1:** Comparison of general characteristics between the 2 groups.

Variable	All	Non-POD group	POD group	
	N = 446	N = 367	N = 79	*P*.overall
Age	69.98 ± 5.72	68.78 ± 5.25	75.59 ± 4.27	<.001
Sex				
Male	188 (42.15%)	153 (41.69%)	35 (44.30%)	.763
Female	258 (57.85%)	214 (58.31%)	44 (55.70%)	
Standard of culture				
Illiteracy	39 (8.74%)	30 (8.17%)	9 (11.39%)	.027
Primary school	134 (30.04%)	102 (27.79%)	32 (40.51%)	
Junior high school or technical secondary school	217 (48.65%)	183 (49.86%)	34 (43.04%)	
High school or college or above	56 (12.56%)	52 (14.17%)	4 (5.06%)	
BMI				
<24	169 (37.89%)	138 (37.60%)	31 (39.24%)	.885
≥24	277 (62.11%)	229 (62.40%)	48 (60.76%)	
History of sleep disorders				
No	367 (82.29%)	313 (85.29%)	54 (68.35%)	.001
Yes	79 (17.71%)	54 (14.71%)	25 (31.65%)	
Mode of living				
Live alone	333 (74.66%)	283 (77.11%)	50 (63.29%)	.016
Live with family	113 (25.34%)	84 (22.89%)	29 (36.71%)	
Preoperative length of stay	2.36 ± 1.07	2.28 ± 1.08	2.73 ± 0.92	<.001
Total hospital stay	7.10 ± 1.13	6.95 ± 1.13	7.77 ± 0.86	<.001
ASA				
I to II	352 (78.92%)	312 (85.01%)	40 (50.63%)	<.001
III to IV	94 (21.08%)	55 (14.99%)	39 (49.37%)	
Combined with hypertension				
No	389 (87.22%)	324 (88.28%)	65 (82.28%)	.206
Yes	57 (12.78%)	43 (11.72%)	14 (17.72%)	
Combined with diabetes				
No	380 (85.20%)	323 (88.01%)	57 (72.15%)	.001
Yes	66 (14.80%)	44 (11.99%)	22 (27.85%)	
Complicated with coronary heart disease				
No	399 (89.46%)	334 (91.01%)	65 (82.28%)	.037
Yes	47 (10.54%)	33 (8.99%)	14 (17.72%)	
Complicated stroke				
No	391 (87.67%)	333 (90.74%)	58 (73.42%)	<.001
Yes	55 (12.33%)	34 (9.26%)	21 (26.58%)	
Combined with COPD				
No	413 (92.60%)	351 (95.64%)	62 (78.48%)	<.001
Yes	33 (7.40%)	16 (4.36%)	17 (21.52%)	
Hypoxemia				
No	380 (85.20%)	331 (90.19%)	49 (62.03%)	<.001
Yes	66 (14.80%)	36 (9.81%)	30 (37.97%)	
Preoperative albumin levels				
Normal	407 (91.26%)	342 (93.19%)	65 (82.28%)	.004
Abnormal	39 (8.74%)	25 (6.81%)	14 (17.72%)	
Hypotension				
No	421 (94.39%)	350 (95.37%)	71 (89.87%)	.062
Yes	25 (5.61%)	17 (4.63%)	8 (10.13%)	
Blood transfusion				
No	413 (92.60%)	345 (94.01%)	68 (86.08%)	.027
Yes	33 (7.40%)	22 (5.99%)	11 (13.92%)	
Preoperative electrolyte				
Normal	414 (92.83%)	341 (92.92%)	73 (92.41%)	1.000
Abnormal	32 (7.17%)	26 (7.08%)	6 (7.59%)	
Anesthesia method				
General anesthesia	419 (93.95%)	347 (94.55%)	72 (91.14%)	.295
Else	27 (6.05%)	20 (5.45%)	7 (8.86%)	
Peroperative bleeding	67.23 ± 19.63	67.04 ± 19.64	68.10 ± 19.67	.664
Anesthesia duration	115.23 ± 7.81	115.11 ± 7.59	115.77 ± 8.76	.496
Operation duration	59.69 ± 5.84	58.63 ± 5.29	64.61 ± 5.77	<.001
Postoperative pain score				
<4	295 (66.14%)	270 (73.57%)	25 (31.65%)	<.001
≥4	151 (33.86%)	97 (26.43%)	54 (68.35%)	
Take sleep aids				
No	420 (94.17%)	347 (94.55%)	73 (92.41%)	.433
Yes	26 (5.83%)	20 (5.45%)	6 (7.59%)	
Use pipertidine after surgery				
No	399 (89.46%)	333 (90.74%)	66 (83.54%)	.092
Yes	47 (10.54%)	34 (9.26%)	13 (16.46%)	

#### 3.2.2. Multivariate analysis

The occurrence of POD was used as the dependent variable, and 16 variables that were found to be closely associated with the occurrence of POD in the univariate analysis were included as independent variables for the unconditional logistic regression analysis. The results revealed that the following 8 variables were independent risk factors for the occurrence of POD after TKA, and they were included in the regression equation: age, educational level, ASA grade [III–IV], stroke history [yes], accompaniment of COPD, postoperative hypoxemia, operation time, and postoperative pain [≥4] (Table 2).

**Table 2:** Multivariate logistic regression analysis of risk factors for POD in older patients after TKA in the 2 groups.

Variable	β	SE	Wald χ^2^	*P*	OR	Lower	Upper
Constant (quantity)	−33.668	5.564	−6.050	<.001	0.000	0.000	0.000
Age	0.241	0.049	4.863	<.001	1.272	1.154	1.401
Educational level	−0.760	0.359	−2.118	.034	0.468	0.232	0.945
ASA	1.597	0.551	2.896	.004	4.936	1.676	14.540
Accompaniment of stroke	1.452	0.617	2.352	.019	4.270	1.274	14.316
Accompaniment of COPD	2.759	0.852	3.237	.001	15.790	2.971	83.925
Hypoxemia	2.326	0.592	3.931	<.001	10.240	3.211	32.658
Operation duration	0.216	0.056	3.853	<.001	1.242	1.112	1.386
Postoperative pain score	1.390	0.503	2.765	.006	4.015	1.499	10.757

### 3.3. Nomograms

**Figure 1.**
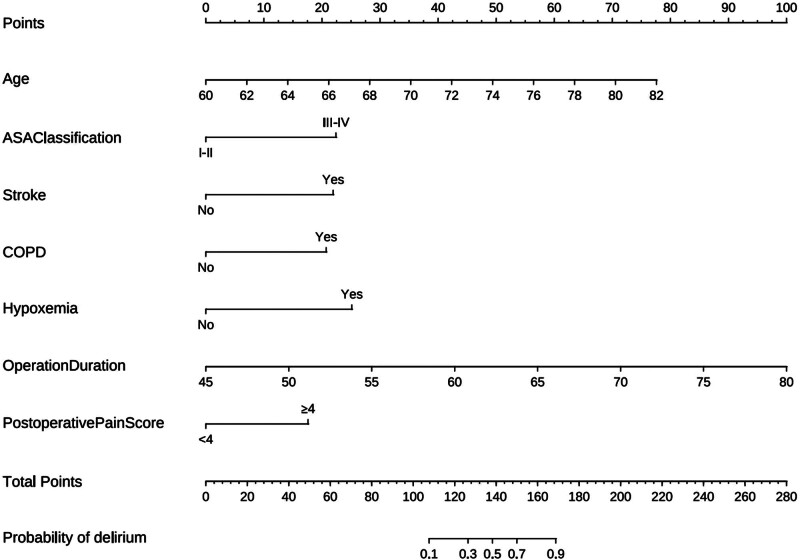
Nomogram of POD in older patients after TKA. POD = postoperative delirium, TKA = total knee arthroplasty.

The prediction model was established according to the results of the regression analysis as follows: Legit (*P*) = −33.67 + 2.759 × COPD + 2.326 × hypoxemia (Yes) + 1.597 × ASA (Yes) + 1.452 × stroke history (Yes) + 1.390 × pain (>4) + 0.241 × age-0.760 × educational level + 0.216 × operation time. The nomogram was also plotted (Fig. 1).

### 3.4. Validation and performance of nomograms

**Figure 2.**
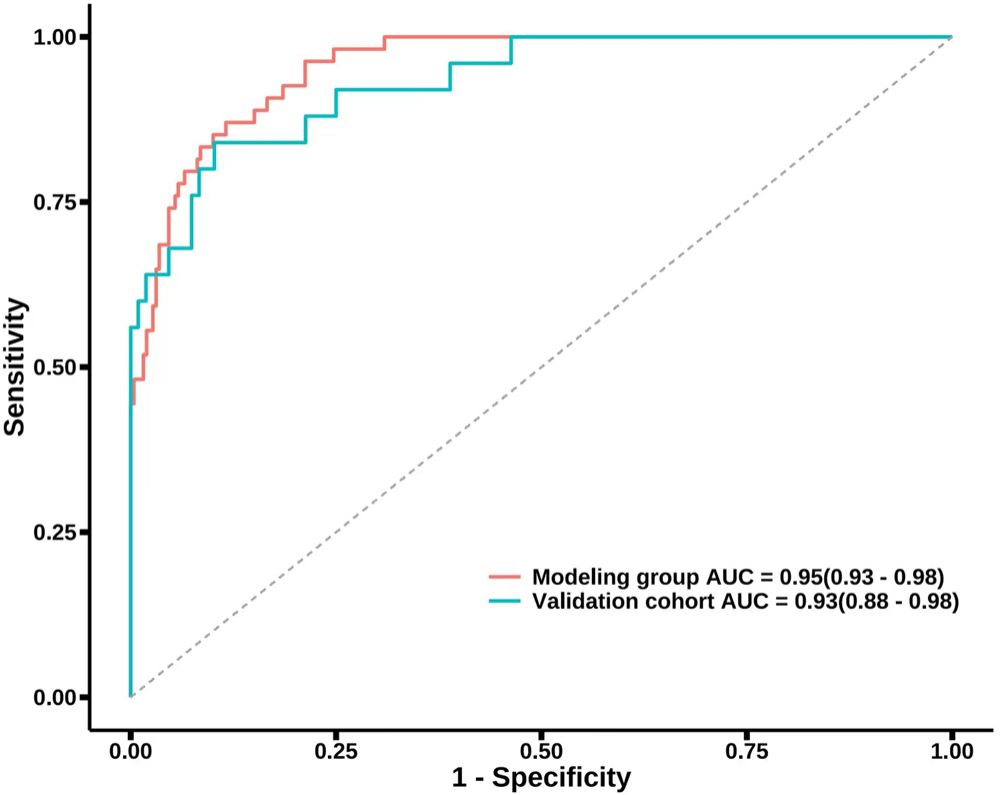
ROC curve of the POD prediction model in older patients after TKA. POD = postoperative delirium, ROC = receiver operating characteristic curve, TKA = total knee arthroplasty.

**Figure 3.**
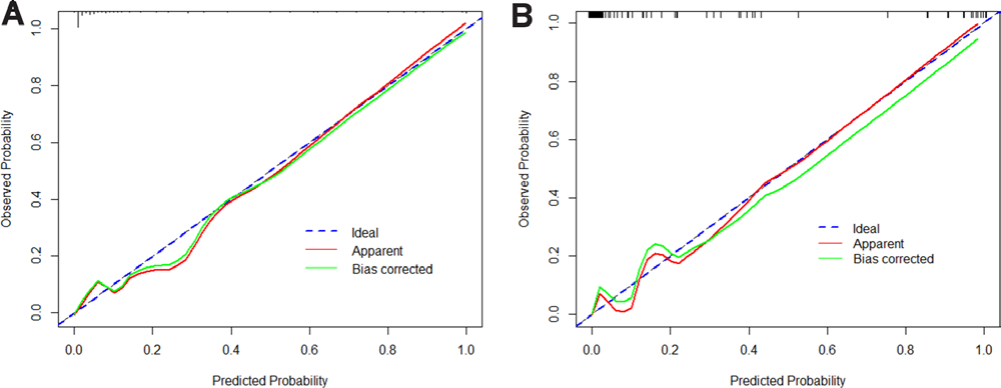
Calibration curve of the POD prediction model in older patients after TKA. (A) Calibration curve of the nomogram in the model group; (B) Calibration curve of the nomogram in the verification group. POD = postoperative delirium, TKA = total knee arthroplasty.

The area under the curve (AUC) was 0.954 (95% confidence interval (CI): 0.93–0.98) in the model group, and the sensitivity, specificity, and Youden index were 0.871, 0.884, and 0.754, respectively. The AUC was 0.931 (95% CI: 0.88–98) in the verification group, and the correct rate of prediction was 91.8%, indicating that the model had a high discrimination capability in predicting the risk of POD occurrence in older patients after TKA (Fig. 2). The results of the Hosmer–Lemeshow goodness-of-fit test in the model group and verification group showed that *P* values were 0.918 and 0.957, respectively (both *P* > .05), demonstrating that the model had a high goodness-of-fit, and it had a high consistency between the predicted risk of POD and actual risk of POD in older patients after TKA (Fig. 3).

## 4. Discussion

### 4.1. It is practical to establish the risk prediction model for POD after TKA

Delirium is a reversible cognitive impairment that may be accompanied with the acute loss of attention and cognitive impairment. The occurrence of delirium is a complex process with multiple factors involved. Older patients undergoing TKA are at the high risk of delirium, and the incidence of POD is as high as 16.23% to 48.1%.^[10,11]^ Multiple studies have shown that the occurrence of POD can prolong the hospital stay and increase the medical costs, and thus, it can not only increase the financial pressure on patients, but also delay the postoperative joint function recovery, resulting in poor prognosis.^[12,13]^Therefore, clarifying the risk factors for the occurrence of POD and developing the risk prediction model for POD after TKA may assist clinicians in implementing targeted preventions, as well as improving the quality of perioperative care.

### 4.2. Analysis of risk factors for POD after TKA

The findings of the present study showed that age, ASA grade, cerebral stroke, postoperative hypoxemia, long operation time, and postoperative pain were independent risk factors for the occurrence of POD after TKA (*P* < .05). The reasons could be summarized as follows: older age: multiple studies have proved that older age is an independent influential factor for POD. With the increase of age, the patient’s brain tissue gradually ages and shrinks, cerebral cortex function decreases, and the number of nerve cells in the brain progressively reduces. In addition, the insufficient cerebral perfusion in older patients can easily induce hypoxia of brain nerve cells. Furthermore, traumas from major surgeries and use of drugs could lead to brain function impairment.^[6,12,14,15]^ Additional attention should be paid to older patients undergoing TKA, in which active and effective measurements should be performed to improve patients’ physical functions, and to minimize the occurrence of POD induced by surgery, drugs, and psychiatric factors; The more educated the patient, the less likely he was to develop delirium. This may be related to patients’ lack of understanding of surgery and anesthesia and excessive fear; high ASA grade: with the elevation of ASA grade, surgery-induced risk and the possibility of brain damage may also increase in patients. Therefore, using active preoperative nutritional support to improve patients’ poor states, timely treatments should be conducted to treat patients’ comorbidities and improve patients’ responses to surgery^[12]^; accompaniment of cerebral stroke: cerebral stroke can induce neuronal damage and death, which affects the vision and brain activities, and induces the cognitive impairment. Therefore, the incidence of POD is high, and the results of the present study are consistent with the most of previous studies.^[15,16]^ For patients with cerebral stroke, cognitive behavioral intervention should be strengthened, and effective measures should be taken actively to reduce the incidence of POD; accompaniment of COPD: Analysis of the reasons may be, patients with a history of COPD induced by surgery, anesthesia and other reasons lead to weakened respiratory function of patients, insufficient oxygen supply to the brain, resulting in delirium; postoperative hypoxemia: hypoxia can aggravate brain tissue stress and cerebral hypoperfusion, inducing chronic ischemia, hypoxia, and edema of brain tissues, resulting in the reduced metabolism, in which patients’ slow thinking and response may be attained, and the risk of delirious is even noteworthy, thereby finally inducing POD.^[17–19]^ In clinical practice, adequate oxygen therapy should be timely given to elevate blood oxygen saturation and improve the state of cerebral hypoxia in patients; long operation time: a longer operation time is associated with the higher risk of adverse effects, including intraoperative hypotension and hypoxemia, causing ischemic and hypoxic damages to brain tissues, resulting in the increased probability of POD^[20,21]^; postoperative pain: postoperative pain may increase the patient’s need for analgesics, and the use of opioids may also lead to delirium while reducing pain, as well as negative emotions (e.g., anxiety and nervousness), which may even cause sleep disturbance in patients, thereby inducing the occurrence of POD.^[22,23]^

### 4.3. Establishing the risk prediction model for POD after TKA appeared advantageous for clinical decision-making

In the present study, a prediction model was established based on the 8 independent risk factors for POD in older patients after TKA, and the results of logistic regression analysis were quantified, graphed, and visualized in the nomogram. It was revealed that a statistical model could assist medical staff to more intuitively evaluate the weights of influences of various factors on POD in older patients after TKA. In the present study, the prediction performance of the model was evaluated by the ROC curve and calibration curve, and the AUC was 0.954 in the model group, and the sensitivity and specificity were 0.871 and 0.884, respectively. In the verification group, the AUC was 0.931, and the rate of correct prediction was 91.8%, indicating that the model had a high degree of discrimination in predicting the risk of POD after TKA in older patients. The results of the Hosmer–Lemeshow goodness-of-fit test in the model group and verification group showed that *P* values were .918 and .957, respectively, indicating that the model had a high goodness-of-fit, and there was a consistency between the predicted risk and the actual risk of POD in older patients after TKA. Therefore, medical staff can quickly screen high-risk groups through the nomogram and develop targeted therapies.

## 5. Conclusion

In conclusion, age, educational level, ASA grade, accompaniment of cerebral stroke, COPD, postoperative hypoxemia, long operation time, and postoperative pain were found as independent risk factors for the occurrence of POD in older people after TKA. The risk prediction model exhibited a promising predictive performance, and clinicians can quickly screen high-risk groups and formulate targeted therapies using the nomograms.

## Author contributions

**Formal analysis:** Ling-Xia Song, Yi Qin.

**Visualization:** Ling-Xia Song.

**Writing – original draft:** Ling-Xia Song.

**Conceptualization:** Yi Qin, Lian-Hong Wang.

**Methodology:** Yi Qin, Ying Li.

**Software:** Yi Qin.

**Data curation:** Li Yang, Ding-Bi Xing, Fu-Qi Lei.

**Validation:** Li Yang.

**Supervision:** Lian-Hong Wang.
